# Care cascade structural intervention versus standard of care in the diagnosis and treatment of HIV in China: a cluster-randomized controlled trial protocol

**DOI:** 10.1186/s12913-017-2323-z

**Published:** 2017-06-12

**Authors:** Yurong Mao, Zunyou Wu, Jennifer M. McGoogan, David Liu, Diane Gu, Lynda Erinoff, Walter Ling, Paul VanVeldhuisen, Roger Detels, Albert L. Hasson, Robert Lindblad, Julio S. G. Montaner, Zhenzhu Tang, Yan Zhao

**Affiliations:** 1 The National Centre for AIDS/STD Prevention and Control, Chinese Center for Disease Control and Prevention, 155 Changbai Road, Changping District, Beijing, 102206 China; 2 National Institute on Drug Abuse, US National Institutes of Health, Bethesda, USA; 3 Integrated Substance Abuse Programs, University of California at Los Angeles (UCLA) School of Medicine, Los Angeles, USA; 4 Emmes Corporation, Rockville, USA; 5 Department of Epidemiology, UCLA Fielding School of Public Health, Los Angeles, USA; 6 British Columbia Centre for Excellence in HIV/AIDS, University of British Columbia, Vancouver, Canada; 7 Guangxi Centre for Disease Control and Prevention, Nanning, China

**Keywords:** HIV test, Point-of-care, CD4 count, Viral load, Viral suppression, Antiretroviral therapy, Mortality, Linkage to care, HIV care cascade, HIV continuum of care

## Abstract

**Background:**

The high rate of attrition along the care cascade of infection with human immunodeficiency virus (HIV) results in lost opportunities to provide timely antiretroviral therapy (ART) and to prevent unnecessarily high mortality. This study aims to assess the effectiveness of a structural intervention, the one-stop (“One4All”) strategy that streamlines China’s HIV care cascade with the intent to improve testing completeness, ART initiation, viral suppression, and mortality.

**Method:**

A two-arm, cluster-randomized controlled trial was implemented in twelve county hospitals in Guangxi China to test the effectiveness of the One4All strategy (intervention arm) compared to the current standard of care (SOC; control arm). The twelve study hospitals were selected for homogeneity and allocated one-to-one to the intervention and control arms. All patients screening HIV positive in study hospitals were enrolled. Target study enrollment was 180 participants per arm, 30 participants per hospital. Basic demographic information was collected as well as HIV risk behavior and route of infection. In intervention hospitals, patients then went on to receive point-of-care CD4 testing and in-parallel viral load (VL) testing whereas patients in control hospitals progressed through the usual SOC cascade. The primary outcome measure was testing completeness within 30 days of positive initial HIV screening result. Testing completeness was defined as receipt of all tests, test results, and post-test counseling. The secondary outcome measure was ART initiation (receipt of first ART prescriptions) within 90 days of positive initial HIV screening result. Tertiary outcome measures were viral suppression (≤200 copies/mL) and all-cause mortality at 12 months.

**Discussion:**

We expect that this first-ever, cluster-randomized controlled trial of a bundle of interventions intended to streamline the HIV care cascade in China (the One4All strategy) will provide strong evidence for the benefit of accelerating diagnosis, thorough clinical assessment, and ART initiation via an optimized HIV care cascade. We furthermore anticipate that this evidence will be valuable to policymakers looking to elevate China’s overall HIV/AIDS response to meet the UNAIDS 90-90-90 targets and the broader, global goal of eradication of the HIV/AIDS epidemic.

**Trial registration:**

ClinicalTrials.gov #NCT02084316. (Registered on March 7, 2014)

## Background

In both resource-rich and resource-limited settings alike, patients are commonly lost at each step along the care cascade of infection with human immunodeficiency virus (HIV) that causes acquired immunodeficiency syndromes (AIDS) [1]. In China, the current standard-of-care (SOC) patient pathway from screening HIV-positive to initiating antiretroviral therapy (ART) involves multiple hospital visits, during which patients submit to several separate blood draws, and then experience protracted waiting periods before they are notified of results. This causes substantial loss to follow-up, delays in diagnosis, clinical assessments that are incomplete, and ART initiation at later stages of disease progression [2, 3]. Recent studies have indicated that, in some parts of China, only approximately 43% of those who screen HIV-positive in hospital settings received confirmatory testing [3], and only 57% of those confirmed HIV-positive received CD4 testing within 6 months [4]. Since CD4 count has been used to determine eligibility for ART, it has been estimated that nearly 80% of those with newly-identified HIV infection, who were ART-eligible, were not initiated on ART in a timely fashion. Unfortunately, these missed opportunities for engagement in the HIV care cascade has ultimately translated to preventable high mortality [2, 5].

Although it seems obvious, it is critically important that people living with HIV (PLHIV) step through all the milestones on the HIV care cascade in order for to fully realize the benefits of ART—they must become aware of their HIV-positive status, receive a diagnosis and clinical assessment, attend counseling, enroll in care and initiate ART, and adhere to their treatment regimens and follow-up schedules [1, 6]. However, merely accessing these services, and sequentially stepping through the process, may not be enough. Increasing evidence has highlighted the importance of the timing in which these services are accessed. Two large, recently-completed clinical trials, TEMPRANO and START, have provided evidence of benefit in not waiting to treat HIV infection until evidence of disease progression is present in the form of symptoms or low CD4 count [7, 8]. This benefit of reduced clinical disease progression, as well as decreased overall mortality, has since been confirmed in other settings, including China [2]. In response, the World Health Organization (WHO) modified its guidelines in 2015, recommending immediate ART initiation for all PLHIV regardless of symptoms or CD4 count [9].

The strong evidence for the clinical benefit of immediate ART for PLHIV [7, 8], taken together with the previously-identified public health benefit in the form of reduced transmission rates [10, 11], meant that optimization of the HIV care cascade was even more important than previously thought. A number of studies in a variety of settings have been designed to examine a range of interventions intended to improve the HIV care cascade. Thus far, majority of these types of studies in low- and middle-income settings have been in Sub-Saharan Africa [12]. However, in general, they have not followed participants beyond the pre-ART period and have not evaluated the effectiveness of multiple interventions implemented as a package [13–19]. One such study has recently been completed and published in China, providing evidence that a simplified test-and-treat intervention can improve both HIV care cascade retention and time to ART initiation [2].

### Objective and outcomes

The objective of the present clinical trial was to evaluate a streamlined pathway for patients from screening HIV-positive to initiating ART, testing the effectiveness of a structural intervention—called the one-stop (“One4All”) strategy. This experimental strategy consisted of a new algorithm incorporating rapid, point-of-care (POC) HIV screening and CD4 testing, and in-parallel plasma viral load (VL) testing to promote fast and complete HIV diagnosis and staging, followed by immediate counseling and ART initiation for eligible patients. The One4All intervention was compared to the current SOC (control arm), and four outcomes were assessed:

1. Testing completeness within 30 days of HIV-positive screening (primary)
2. ART initiation within 90 days of HIV-positive screening (secondary)
3. Viral suppression (≤200 copies/mm^3^) at 12 months after HIV-positive screening (tertiary)
4. All-cause mortality at 12 months after HIV-positive screening (tertiary)

## Method

### Design

**Fig. 1:**
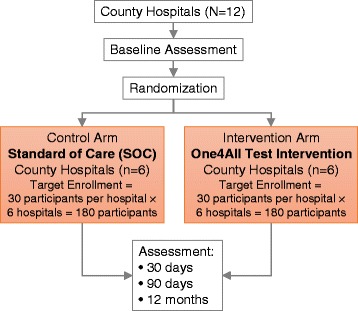
Diagram of study design. This diagram illustrates the cluster-randomized controlled study design with one-to-one allocation of twelve county-level hospitals to either the control arm (current standard of care [SOC]) or the intervention arm (the “One4All” strategy intended to streamline the HIV care cascade)

A cluster-randomized trial was designed as diagrammed in Fig. 1. Hospitals were the unit of randomization and twelve similar county-level hospitals were allocated one-to-one to the intervention and control arms of the study. The efficacy of the intervention was determined by comparison to the current standard of care (SOC, control arm) at 30 days, 90 days, and 12 months.

### Setting

**Fig. 2:**
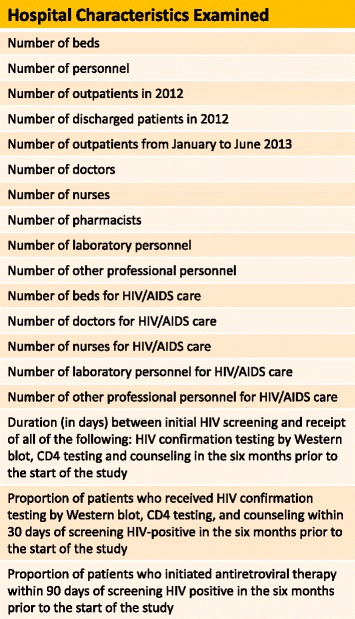
List of Hospital Characteristics. The eighteen different characteristics listed here were examined in the process of assessing candidate county-level hospitals in Guangxi, China. The final twelve study hospitals were selected based upon homogeneity across these characteristics

The setting for this study was county hospitals in Guangxi Zhuang Autonomous Region (hereafter referred to as Guangxi). In 2011, Guangxi reported the second highest cumulative number of HIV/AIDS cases in China, the highest number of newly-reported HIV/AIDS cases, the highest number of newly-reported AIDS cases, and the highest number of AIDS-related deaths [5]. As shown in Fig. 2, a broad range of characteristics of potential county-level hospitals in Guangxi were examined. The hospitals included in the study were selected based on their homogeneity across these characteristics.

### Allocation and blinding

The twelve selected study hospitals were stratified by historical rates of testing completeness followed by post-test counseling during the first six months of 2013 prior to being allocated to the intervention or control study arms. Eight hospitals were within the low historical testing completion stratum of <20%, and these eight hospitals were randomized, four into the intervention arm and four into the control arm. Four hospitals were within the high historical testing completion stratum of ≥20%, and these four hospitals were randomized, two into the intervention arm and two into the control arm. All patients who screened HIV-positive in the six intervention hospitals received the One4All intervention and all patients who screened HIV-positive in the six control hospitals during the same study period received SOC services. There was no blinding required since the unit of randomization was the study hospital and each hospital only offered the HIV care cascade it was assigned during randomization—SOC or One4All.

### Participants and recruitment

Study eligibility criteria were: [a] being 18 years of age or older, [b] having a positive result on an HIV enzyme immunoassay (EIA) screening test, [c] seeking care in a study hospital, either inpatient or outpatient, and [d] residing or intending to reside within the study catchment area. Study exclusion criteria were: [a] having previously received confirmation of HIV infection in any setting, [b] being a prisoner or detainee at time of screening, or [c] being a pregnant woman.

No special strategies to ensure adequate participant enrollment were employed. All patients who were found to be eligible were enrolled in the study. After enrollment, basic demographic information was collected. Information on self-reported risk behavior and route of HIV acquisition were also collected.

### Ethics

The protocol and consent process were reviewed and approved by the US National Institutes of Health (NIH), National Institute on Drug Abuse (NIDA) Protocol Review Board and Data and Safety Monitoring Board, as well as respective institutional review boards (IRBs) of the University of California, Los Angeles (UCLA) and the National Center for AIDS/STD Control and Prevention (NCAIDS), China Center for Disease Control and Prevention (CDC).

Individual informed consent was not obtained. Study information was shared with participants and participants were given opportunities ask questions. While participants could refuse to participate, or to share their data, their enrollment still counted toward the total number of study participants. No study-related reimbursement for participants was provided.

All patient data were protected and kept confidential according to standard procedures present within China’s healthcare system. Participant data extracted from hospital records, and from China’s National HIV/AIDS Comprehensive Response Information Management System (CRIMS, which has been described elsewhere) [20], were assigned trial-specific participant identification numbers and then otherwise de-identified prior to use to protect participant privacy.

### Sample size

A minimum sample size of 180 participants per arm, across 12 clusters (or 30 participants per cluster) was selected. This sample size was expected to achieve 93% power based on a one-sided test (at alpha = 0.05) to detect a difference between the group proportions of 0.28, where under the alternative hypothesis the One4All arm proportion was assumed to be 0.50 and the control arm proportion was assumed to be 0.22. This calculation assumed an intra-class correlation (ICC) within hospitals of 0.082 based on preliminary data. Although 30 participants per cluster (or hospital) was the target, it was expected that some hospitals would enroll participants more quickly than others. Therefore, a final study population of greater than 180 participants per arm was anticipated as enrollment at all hospitals remained open until the last hospital met its 30-participant goal.

### Intervention

**Fig. 3:**
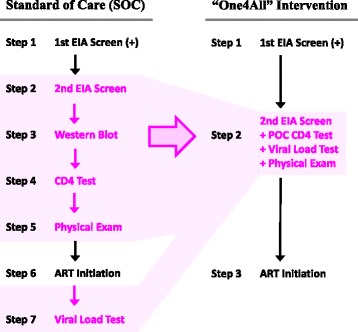
Illustration of Intervention and Control Conditions. This illustration depicts the differences in the HIV care cascade between control (standard of care [SOC]) and intervention (“One4All” strategy) study arms. In the SOC condition, HIV care cascade steps 2, 3, 4, 5, and 7 are all consolidated into a single step 2 in the One4All condition

A high-level overview of the HIV care cascade in SOC (control arm) and One4All (intervention arm) hospitals is depicted in Fig. 3. The One4All test intervention included rapid, POC HIV EIA screening and CD4 testing, with in-parallel VL testing. This strategy was intended to promote rapid and complete diagnostic assessment and accelerate time to ART initiation for those deemed ART eligible. The ART eligibility threshold at the time of this study was CD4 count ≤350 cells/mm^3^. Each of the steps in the HIV care cascade up to assessment of ART eligibility are described in detail below for both the SOC condition and the One4All condition. In both care cascades, all participants received post-test counselling each and every time they received test results.

#### HIV Screening

*In SOC study hospitals,* the Wantai Screening HIV (1 + 2) Ag&Ab EIA (Beijing Wantai Manufacturer of Infectious Diseases Diagnostics) was used as the initial screening test followed by two different EIAs that varied between sites.

*In One4All study hospitals,* the first HIV screening test was the also the Wantai Screening HIV (1 + 2) Ag&Ab EIA. The second and the third EIAs were the Determine HIV-1/2^®^ rapid test (Abbott Laboratories) and the InTec HIV rapid test (Xiameng InTec Products).

#### HIV Confirmation

*In SOC study hospitals,* an additional blood sample was collected immediately following screening or on a subsequent study hospital visit. The sample was sent to the local city CDC laboratory for Western blot (WB) confirmatory testing according to usual practice. As confirmatory testing required approximately 10 to 15 days to be completed, participants typically had to be contacted and asked to return to the study hospital to receive their results.

*In One4All study hospitals,* WB was not used for confirmatory testing. Rather, VL testing served as the confirmatory test (see below).

#### CD4 Count

*In SOC study hospitals,* participants were asked to return to the study hospital for another blood draw for CD4 testing after they were notified that the result of their confirmatory testing was positive. Blood specimens were again sent to the city CDC laboratory, this time for CD4 testing. CD4 testing also required approximately 10 to 15 days to complete. Once CD4 test results were available, participants were again asked to return to be notified.

*In One4All study hospitals,* POC CD4 testing was performed in the study hospital with a POC Pima™ CD4 Analyzer (Alere Healthcare, USA) using whole blood samples. Results were available within 30 min. Participants were notified of their results as soon as possible, but no later than the next day if they had left the study hospital during the test.

#### Viral load

*In SOC study hospitals,* VL testing was conducted approximately one year after ART initiation. Participants were asked to return to the study hospital for blood sample collection. Blood samples were sent to the provincial CDC laboratory for plasma VL testing, which required 10 to 15 days to complete. Participants were contacted and asked to return to the study hospital to receive VL testing results once they were available.

*In One4All study hospitals,* blood samples for VL testing were collected immediately following receipt of positive screening results, at the same time as the blood draw for CD4 testing (see above). VL testing was conducted again after one year. Plasma VL testing itself was conducted in the same manner as in SOC study hospitals—samples were sent to the provincial CDC laboratory, plasma VL testing required 10 to 15 days, and participants asked to return to the study hospital to receive VL test results.

This intervention condition posed no specific safety risk to participants. In comparison to SOC study hospitals, the HIV care cascade in One4All study hospitals involved fewer blood draws and there was no increase in the volume of blood drawn or the typical risk associated with blood draws.

### Outcomes

The primary outcome measure was the proportion of participants who achieved testing completeness and received test results and post-test counseling within 30 days of positive screening. Testing completeness was defined as completion of three required components: 1) initial HIV screening (one to two tests in One4All and two to four in SOC), 2) CD4 testing, and 3) confirmatory HIV testing—WB in the SOC arm, or VL in the One4All arm. “Success” was defined as completion of all three required tests and notification of test result along with receipt of counseling after each test. Success was compared at 30 days after initial HIV-positive screening. The secondary outcome measure was the proportion of participants who initiated ART within 90 days from the date of HIV-positive screening. ART-initiation was defined as the receipt of the first ART prescription. Tertiary outcomes included the proportion of participants who achieved viral suppression (≤200 copies/mm^3^) and mortality at 12 months. Mortality was assessed as the number of reported deaths divided by the number of patients enrolled.

Aside from these main outcome measures, three other measures were pre-specified. For the primary outcome, two further analyses were planned: time from initial positive HIV screen to success, not restricted to the 30-day window, compared between control and intervention arms, and success at 45, 60, and 90 days, also compared between control and intervention arms. For the secondary outcome, one further analysis was planned: time from initial positive HIV screen to ART initiation, not restricted to the 90-day window, compared between control and intervention arms.

### Timeline

**Fig. 4:**
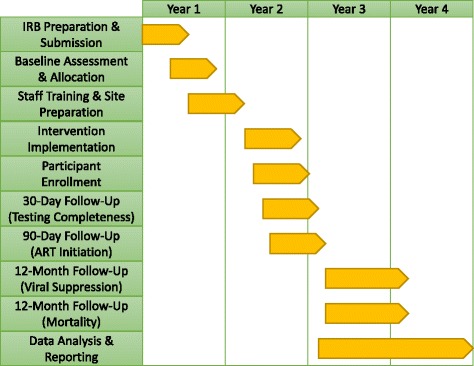
Timeline of the Study. The study was expected to require approximately 14 months for pre-implementation preparation (i.e. IRB preparation, submission, review, and approval; baseline hospital assessment, selection, and allocation; and staff training and site preparation). Implementation and enrollment were expected to take a further 9 months, and follow-up required a subsequent 12-month period. Finally, data analysis and reporting were expected to require another 12 months. (institutional review board [IRB], antiretroviral therapy [ART])

A general study timeline is depicted in Fig. 4. Significant preparation prior to trial commencement was required and included completion of the IRB processes at multiple institutions, baseline hospital assessment, selection, and allocation, and staff training and site preparation. This phase was expected to take approximately 14 months. Once the study began, implementation and enrollment were anticipated to take 9 months, and follow-up a further 12 months. After trial completion, data analysis and reporting was planned for the following 12 months.

### Data collection, management, and monitoring

Baseline assessment information, including participant contact and demographic information, laboratory screening results and dates, test result notification dates, and post-test counseling dates were all collected using case reporting forms (CRFs). CRF data were stored in a web-based database constructed specifically for the study. This database served as the centralized location for electronic storage of all study-related data. Study-related data from each hospital system was retrieved weekly and uploaded to this study database.

To supplement the data contained in the study-specific database, records were extracted from CRIMS [20]. Each county hospital nationwide, including all participating study hospitals, have unique identification codes in CRIMS to facilitate extraction of all data on HIV cases identified at study hospitals.

A centralized Data and Statistics Team (DST) was responsible for the validation of the web-based study database, retrieval of study hospital data, data extraction from CRIMS, merger of the two datasets into one, assurance of data integrity and security, and development and delivery of training for participating hospital and CDC staff members on applicable data management procedures.

### Data analysis

To adjust for both the clustering effect of hospitals (with hospital as a random effect to account for the ICC) and baseline participant-level and hospital-level confounding factors, analysis of the primary outcome measure was performed using G-side GLIMMIX modeling. Participant-level factors included in the model were age, gender, ethnicity, education, occupation, marital status, transmission route, and treatment setting (inpatient/outpatient), and hospital-level factors included baseline test completion rate prior to randomization. To assess the consistency of the primary outcome results, a Chi-square test adjusted for clustering was also used to measure the association between treatment arm and the primary outcome [21], although this method does not account for baseline confounders. Secondary analyses of the primary outcome measure were performed as a waiting time analysis examining time from initial positive screen to success. For this waiting time analysis, participants who did not meet the testing success criteria were censored at their last follow-up dates. Kaplan-Meier curves were used to display differences over time from screening to testing and counseling completeness.

In comparing differences in baseline characteristics between the two arms, a Chi-square test adjusted for clustering was performed for binary variables. When baseline categorical variables had more than 2 responses, an adjusted *p*-value was calculated by referring the observed (unadjusted) Chi-square value to the distribution of Chi-square values obtained by randomly permuting observed treatments over hospitals, with the adjusted *p*-value defined as the proportion of 'permuted' Chi-square values at least as large as observed. To test between-arm differences in continuous data, a *t*-test adjusted for clustering was performed [21].

Analytical methods and the adjustment for clustering and covariates effects used in the analysis of the primary outcome were also applied to the ART initiation secondary outcome and viral suppression tertiary outcome. As with the secondary analysis of the primary outcome, Kaplan-Meier analyses were also performed for time to initiation of ART, outside the 90-day window, and time to death. In addition, for the mortality outcome, a random effects Cox (shared frailty) model accounting for the hospital clustering effect was used to calculate the intervention effect on the hazard ratio (HR) of death while also controlling for other covariates. Adjustments to *p*-values and confidence intervals for multiple testing and multiple comparisons were not performed. All data analyses were performed using SAS software (SAS Institute, Inc., USA).

### Dissemination

The trial is registered with ClinicalTrials.gov (#NCT02084316) and the original protocol has been published on CTNDisseminationLibrary.org. The dataset is available upon request to the corresponding author.

## Discussion

To our knowledge this is the first controlled clinical trial to examine a bundle of interventions intended to improve multiple points in China’s HIV care cascade. A notable strength of this study lies in the rigor with which the effectiveness of the One4All strategy was tested. We expected that it would provide strong evidence in support of a streamlined HIV care cascade that accelerates diagnosis, complete clinical assessment, and treatment initiation having a significant clinical benefit over the current SOC.

With growing global evidence of the clear benefits of immediate ART for all PLHIV, both for the purposes of treatment of HIV infection and of prevention of HIV transmission [7, 8, 10], HIV/AIDS programs around the world must place renewed focus on optimization of the HIV care cascade. Worldwide, three out of every five PLHIV have not accessed ART [22]. Steps must be taken to eliminate losses to follow-up in the pre-ART period, and to ensure that all PLHIV receive immediate treatment.

At the time the present study was conducted, China still had a CD4 count ≤350 cells/mm^3^ ART eligibility criterion in place, with exceptions allowed only for PLHIV who were pregnant or in serodiscordant relationships [23] Thus, we expected that some participants in our study could not initiate ART despite being successfully retained in the pre-ART HIV care cascade. The WHO has since elevated the CD4 count threshold for ART to CD4 count ≤500 cells/mm^3^ in 2013 [24], and then abolished it in 2015, now recommending that all PLHIV receive immediate ART regardless of CD4 count [9]. Two studies in China have recently examined the benefit of ART initiation for PLHIV who did not meet the ≤350 cells/mm^3^ threshold. One prospectively examined immediate ART for all participants regardless of CD4 count [2], the other retrospectively investigated ART given to PLHIV with CD4 counts >500 cells/mm^3^ on an exception basis or as a part of the NCAIDS-sponsored “Prevention and Treatment of Major Infectious Diseases” project launched in 2012, which consisted of a series of cluster-randomized controlled trials among key populations. Both studies found significant improvements in mortality. We envision the combination of this evidence with the outcome of the present study supporting the complete replacement of China’s current SOC HIV care cascade with a new, streamlined cascade that accelerates diagnosis, complete clinical assessment, and ART initiation for all PLHIV.

Several important challenges were anticipated prior to trial commencement. For example, there was known variability in HIV SOC offered between different counties in Guangxi. A major cause of poor testing completeness and treatment initiation in rural Guangxi is disjointed HIV services as a result of structural and cultural constraints. County hospitals and CDCs struggle to follow national guidelines, mainly because of limited laboratory capacity and complex referral systems between hospitals and CDCs. The study team debated whether to provide strict guidelines and training to control arm study hospitals in an effort to standardize their current practice and improve homogeneity. However, we ultimately decided against tampering with the control condition, as we determined that a control comparator that represented the reality of the current standard HIV care cascade was more valuable than an artificial ideal control environment we would have created only for the benefit of the study. Therefore, during our baseline hospital investigations, the investigators engaged with hospital leaders and key personnel involved in HIV care and collected the key characteristics of the routine practice adopted at each county. A detailed testing to treatment flowchart was developed for each of the hospitals, documenting the key deviations from national guidelines. Then, CRFs were adjusted in order to accurately reflect the reality of testing and linkage to care procedures in each hospital.

Another key challenge was in the size of the study. The study was originally designed to include 24 hospitals (or clusters), 12 each in the control and intervention arms. This would have provided adequate power to detect differences between arms in all four outcomes. However, due to both the known high degree of variability observed in SOC services across counties in Guangxi and historically low numbers of patients screening HIV positive in many hospitals, not to mention the inherent difficulties with implementing such a large study, we decided to scale back the number of hospitals to 12. Although we suspected that we would still find statistically significant differences in several of the outcomes, the study was truly only adequately powered to detect between-arm differences in the primary outcome of testing completeness by 30 days.

As the study included no intervention related to ART regimen adherence, we anticipated the possibility that we may find no between-arm differences in achievement of viral suppression. We chose to define viral suppression as ≤200 copies/mL based on the equipment used for the study in local laboratories, recognizing that this target was aggressive. Although above the <50 copies/mL definition of viral suppression commonly recognized in high-income settings, it is well below the <1,000 copies/mL threshold recognized by the WHO as achievement of viral suppression in low- and middle-income countries such as China. It is also below the 300 to 500 copies per/mL target commonly used by research teams in studies in low- and middle-income settings [1, 25]. Future studies will need to examine interventions aimed at improving adherence in order to promote achievement of viral suppression. Additionally, with increased numbers of PLHIV on ART, it will be necessary for China’s public health and healthcare communities to be vigilant in monitoring patients for treatment failure. This will require substantial improvement of VL testing infrastructure and China should strongly consider investment in the development and implementation of new POC VL testing technologies, which further contribute to a streamlined HIV care cascade.

## Conclusion

This first-ever, cluster-randomized controlled trial of a bundle of interventions intended to streamline the HIV care cascade in China (the One4All strategy) was evaluated on four outcomes: testing completeness within 30 days, ART initiation within 90 days, viral suppression at 12 months, and all-cause mortality at 12 months. We expect that the outcome of this trial will provide strong evidence for the benefit of accelerating diagnosis, thorough clinical assessment, and ART initiation via an optimized HIV care cascade such as the One4All strategy. Furthermore, we anticipate that this evidence taken together with the strong, new evidence of both treatment and prevention benefits of immediate ART for all PLHIV regardless of CD4 count [7, 8, 10], will compel careful consideration by policymakers in China and other, similar low- and middle-income countries. HIV care cascade optimization is a critical strategy for meeting the UNAIDS 90-90-90 targets [26], and will be required for the eventual eradication of the HIV epidemic.

## Abbreviations

AIDS: Acquired immunodeficiency syndromes
ART: Antiretroviral therapy
CDC: Center for disease control and prevention
CRF: Case reporting form
CRIMS: Comprehensive response information management system
DST: Data and statistics team
EIA: Enzyme immunoassay
HIV: Human immunodeficiency virus
HR: Hazard ratio
ICC: Intra-class correlation
IRB: Institutional review boards
NCAIDS: National Center for AIDS/STD Control and Prevention
NIDA: National Institute on Drug Abuse
NIH: National Institutes of Health
One4All: The one-stop strategy intervention
PLHIV: People living with HIV
POC: Point-of-care
SOC: Standard-of-care
UCLA: University of California, Los Angeles
UNAIDS: The Joint United Nations Programme on HIV/AIDS
VL: Viral load
WHO: World Health Organization
